# Unusual foreign body in a teenage boy: Case presentation and review of the literature

**DOI:** 10.1002/jpr3.70008

**Published:** 2025-02-23

**Authors:** Maria Misiou, Antonia Jeličič Kadič, Matjaž Homan

**Affiliations:** ^1^ Department of Paediatris University Hospital of Larisa Thessaly Greece; ^2^ Department of Paediatrics University Hospital of Split Split Croatia; ^3^ Department of Gastroenterology, Hepatology, and Nutrition, University Children's Hospital, Faculty of Medicine University of Ljubljana Ljubljana Slovenia

**Keywords:** bottle, endoscopy, rectum

## Abstract

Foreign bodies (FBs) in the lower gastrointestinal tract are sporadically described in children. The therapeutic approach is individualized, depending on the type of FB, the location, time since insertion and the severity of bowel injuries. These FBs can be frequently removed manually or endoscopically. However, exploratory laparotomy is inevitable in unsuccessful and complicated cases. Herein we present a teenager boy with a self‐inserted large perfume bottle in the sigma, and we performed the review of the published literature.

## 1 INTRODUCTION

Rectal foreign body (FB) is a challenging condition, starting with the emergency department examination and continuing to the postextraction stage. In adults, it is a well‐described phenomenon with a rising incidence. On the contrary, in the pediatric emergency department, it is a rarely seen condition. FB can be inserted in the rectum for a variety of reasons in children, including exploration, self‐gratification, or sexual abuse.

Approaching the patient is crucial as it should be tailored according to the type, size of foreign object, duration, possible complications, and rectal injuries, especially because there is no well‐established treatment algorithm.

Therefore, we describe a case of an unusual rectal FB in a 14‐year‐old boy and discuss the modality of its removal.

## 2 CASE REPORT

**Figure 1:**
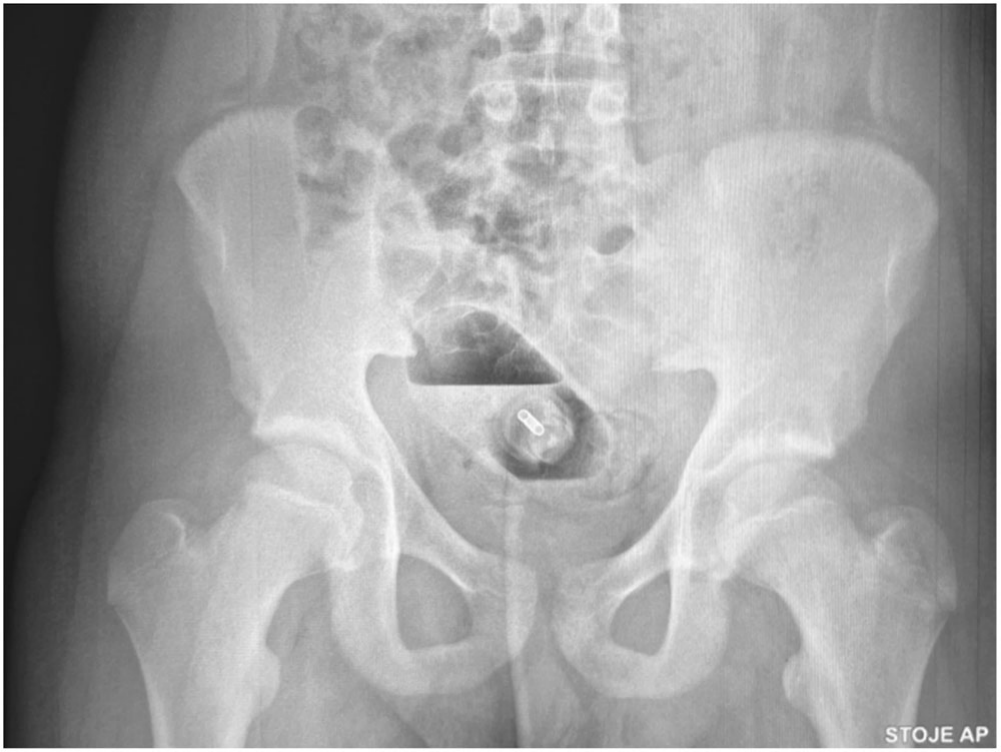
Abdominal x‐ray of the large FB. FB, foreign body.

**Figure 2:**
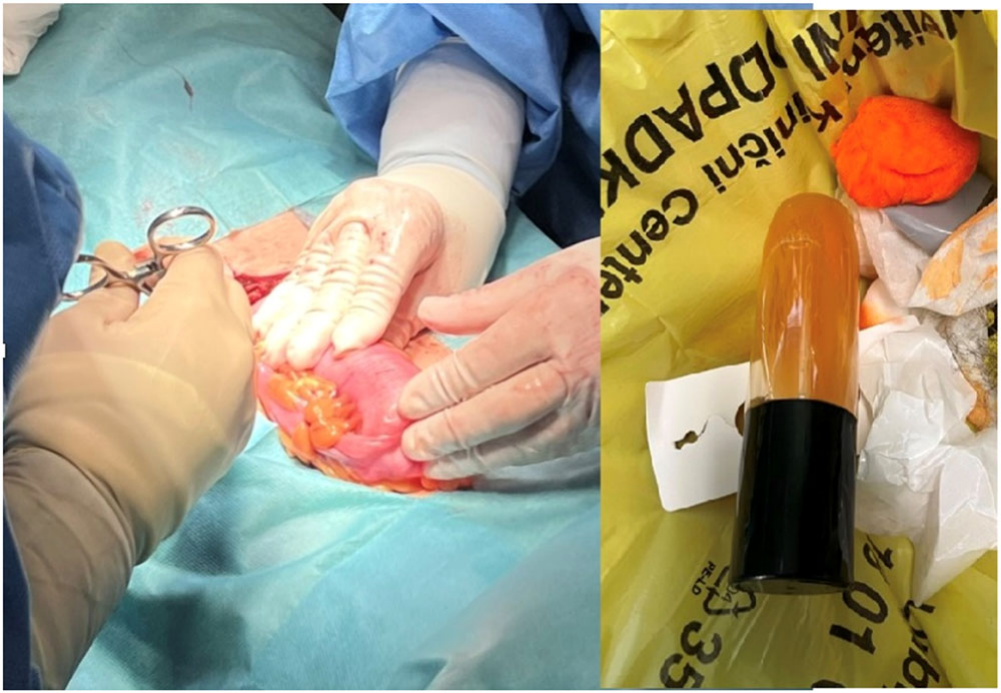
A picture of laparotomy and FB (after removal). FB, foreign body.

A 14‐year‐old boy inserted a large FB (perfume bottle (18 cm × 5 cm), still containing liquids) into his anus for self‐gratification. He was still asymptomatic 36 h later; however, he visited the emergency department of a tertiary hospital accompanied by his mother. His vital signs, physical examination, and basic laboratory tests were normal. There were no visible erosions of anal region or FB in the rectum. A plain abdominal radiograph revealed a large oval object lying in the sigmoid region (Figure 1). Pediatric surgeon on call unsuccessfully tried to extract the bottle manually. The next day, removal with a colonoscope was attempted under general anesthesia by a pediatric gastroenterologist. A large hard glass object was identified 30 cm above the anus. The endoscopist tried to grasp the FB with a large snare and remove it; multiple trials in combination with manual transabdominal manipulation were unsuccessful due to the large size of the FB jammed into the colon incline and especially the slick, nongripable material. Consultation with the pediatric surgical service was again requested, and the patient was transferred directly to the surgery room. Exploratory laparotomy was performed, and the incarcerated FB was clearly visible and palpable through the bowel wall, which was not damaged due to possible ischemia and necrosis. Therefore, the surgeon manually squeezed the FB toward the rectum, where the perfume bottle was pulled out without damaging the rectal wall (Figure 2). After the procedure, the patient went home without any complications.

## 3 DISCUSSION

FBs in the rectum are mostly described in adults. Over 3500 FBs have been removed from the rectum of patients in the United Kingdom in a decade.^1^ In the pediatric population, FBs in the gastrointestinal tract are usually ingested, most often in preschoolers. Cases of FB insertion are rare and mostly refer to case reports.^2^, ^3^ A study by Fischer et al., which characterizes the rectal injuries from FB insertion in children, demonstrates a significant uptrend.^4^ The rising number of FBs in pediatrics may be related to multifactorial causes such as easy internet access with porn sites.

Among the described objects inserted in the rectum are massage devices, vibrators, pens, pencils, ropes, strings, bottles, jars (glass or other material), corncob, gravel, and stylus batteries.^2^, ^4^

In view of the published literature, children can insert objects in their anus for various reasons. First, in their early years and in the context of early sexual exploration and curiosity, they tend to insert objects in their orifices. Later, adolescents insert objects in their anus for self‐gratification, as seen in our case. Previous studies suggested that child abuse can be a cause of rectal FB in young children. Fischer et al., in contrast, revealed that only 1.9% of all rectal FBs were secondary to suspected abuse.^4^ Donaruma‐Kwoh et al.^2^ characterized the physical examination findings in children and adolescents, who disclosed the insertion of an object into their bodies as part of their sexual abuse history. They identified two cases; two male patients aged 5 and 9, respectively, had foreign objects detected in the workup for their complaints of abdominal pain.

A comprehensive approach to the diagnosis and treatment of FBs is necessary due to the large variety of instruments and possible injury produced to the rectum and distal colon. Fischer et al. described austere FB injuries in children and adolescents presenting to emergency departments in the United States from the period of January 2008 to January 2017. This study identified that 88% of rectal FB cases required an escalation of treatment. That occurred more frequently in the rectal than other genitourinary insertions.^4^ Management strategies for such FBs come from adult cases and commonly include transanal extraction, removal under anesthesia, endoscopic retrieval, and various surgical modalities, including minimally invasive surgery, laparoscopic‐assisted surgery, and open surgery.

A retained rectal FB can be classified depending on its location relative to the rectosigmoid junction. Clinically, a low‐lying rectal FB is considered when it is palpable on a digital rectal examination, which could allow its extraction transanally. If unsuccessful, removal could require endoscopy or surgery. Rectal FBs which are retained higher, as it was in our patient, usually requires endoscopic or surgical intervention.^5^ The endoscopic extraction technique is safe and usually very effective. This technique allows visualization of the location of the foreign object as well as the surrounding mucosa, and a polypectomy snare or forceps can be used to help extract the FB. The endoscope can also be used to evaluate for possible mucosal injuries after successful extraction.^6^ Possible limitations for endoscopic removal are the large size and slippery material of FB, making removal of a rectal FB more difficult. The bottle in our case was very large and made of slippery material.

To date, formal clinical guidelines about the management of retained rectal FB in adults are lacking, even though the phenomenon is well described. Some authors^7^, ^8^ suggested bedside extraction in the emergency department as first‐line management. Cawich et al.^6^ suggested the early surgical referral and rectal exam under anesthesia with no attempt to transanally extract the foreign object in the emergency department, due to the high failure rates as the object might be advanced higher into the rectosigmoid region, as happened in our case. We believe the first thing to consider, is which method is less invasive and safest, depending on the experience and skills of medical professionals.

The data from relevant literature about rectal FBs in children are collected in Table 1.

**Table 1:** Literature review of FB insertion in children.

Study	No. of patients	Age (years)	Cause	Symptoms	FBs	Treatment
Bouamama et al.^9^	1	10	NA	Peritonitis	Plastic tube	Laparotomy
Singer and Saxena^3^	1	13	Self‐gratification	No	Vibrator	Enemas
Slingsby and Goldberg^10^	1	12	Self‐exploration	Abdominal pain	Cylindrical tube	Endoscopy
Shao et al.^11^	2	3.6	NA	no	Thermometer	Laparotomy
Fischer et al.^4^	225	0–25	Self‐exploration	NA	Massage devices, vibrators, Rope or string, Pens and pencils, bottles or jars,	Released without treatment, transanal extraction, surgical modalities
Lovisetto et al.^12^	1	17	Asperger syndrome	Abdominal pain	30 stylus batteries	Digital removal/endoscopy
Donaruma‐Kwoh et al.^2^	2	5.9	Sexual abuse	Abdominal pain	Gravel, corncob	Enemas, endoscopy, manually

Abbreviations: FB, foreign body; NA, not applicable.

## CONFLICT OF INTEREST STATEMENT

The authors declare no conflicts of interest.

## ETHICS STATEMENT

Written informed consent was obtained from the patient and family for the publication of this case.
